# Efficacy of Teletherapy vs. Conventional Therapy in Improving Depressive Symptoms: A Meta-Analysis of PHQ-9 Scores Outcomes

**DOI:** 10.1192/j.eurpsy.2025.1425

**Published:** 2025-08-26

**Authors:** S. Queiroz, D. Fernandes de Holanda, I. Salha

**Affiliations:** 1 Medicina, UEPG, Ponta Grossa; 2 Medicina, UFAM, Manaus, Brazil; 3 Medicine, Internation Organization of Migration (IOM), Palestinian, Palestinian, State of

## Abstract

**Introduction:**

Tele-rehabilitation (Tele-TR) has emerged as a promising alternative to traditional, in-person cardiac rehabilitation (Conventional-CR), offering accessible and convenient rehabilitation options for patients who may face logistical or health-related barriers to attending conventional programs. Advances in digital health technologies, coupled with the increasing demand for remote healthcare solutions, have led to the widespread adoption of Tele-TR, particularly for patients recovering from cardiac events or undergoing surgery. However, there is a need for a comprehensive evaluation of the comparative effectiveness of Tele-TR versus Conventional-CR, especially in terms of clinical outcomes. Given the rise in mental health concerns among patients undergoing cardiac rehabilitation, the impact of these rehabilitation modalities on psychological well-being, particularly depressive symptoms, has become an important area of focus.

**Objectives:**

This meta-analysis aims to evaluate the efficacy of Tele-TR in comparison to Conventional-CR, specifically assessing their effects on depressive symptoms. The primary objective is to determine the mean differences in health assessment scores, such as the PHQ-9, across studies that compare Tele-TR and Conventional-CR. By synthesizing the findings from multiple randomized controlled trials (RCTs), this analysis will provide insights into the relative effectiveness of these two rehabilitation approaches, with a particular emphasis on mental health outcomes and their implications for patient recovery and quality of life.

**Methods:**

Three studies were included, comparing Tele-TR with Conventional-CR. Outcomes were assessed based on mean differences (MDs) and 95% confidence intervals (CIs) for continuous data. Heterogeneity among studies was quantified using Tau² and I² statistics, and a random-effects model was employed to account for variability across studies.

**Results:**

The analysis of post-treatment improvement in PHQ-9 scores across included studies demonstrated an overall mean difference of -1.94 (95% CI [-4.02; 0.15]), suggesting a trend favoring Tele-TR over Conventional-CR. However, this improvement did not reach statistical significance (Z = -1.82, P = 0.07). High heterogeneity was noted (Tau² = 3.36, I² = 100%, P < 0.01), indicating substantial variability in treatment effects among studies.

**Image:**

**Figure:**
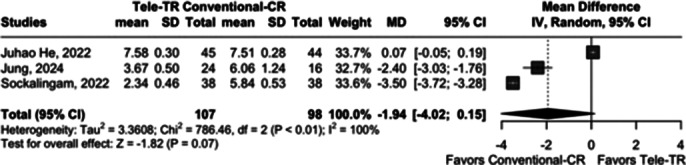

**Image 2:**

**Figure:**
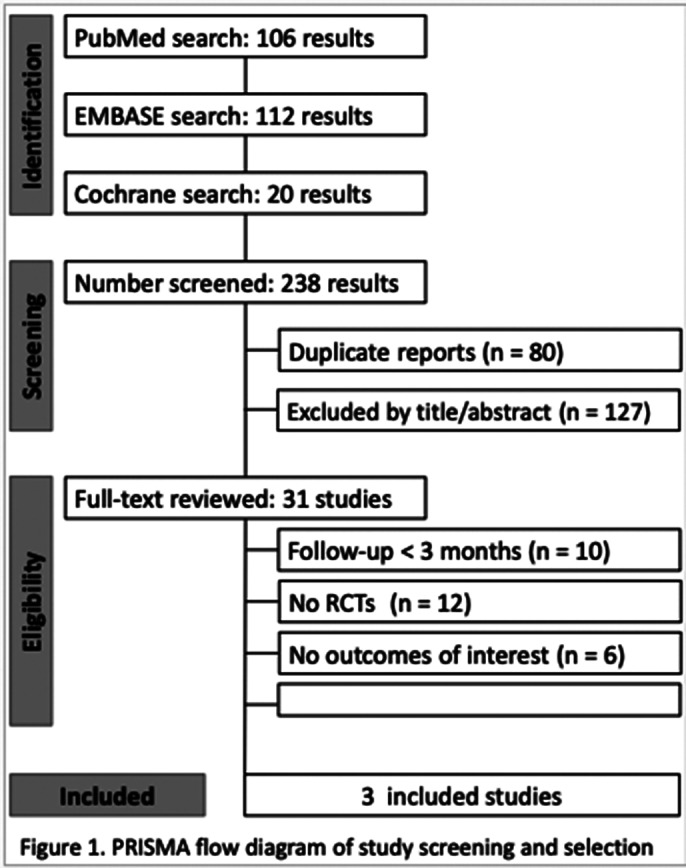

**Conclusions:**

While Tele-TR showed a trend toward improvement over Conventional-CR, the findings did not achieve statistical significance. High heterogeneity suggests differences in study design and patient populations, warranting further research to clarify the potential benefits of Tele-TR and identify the contexts where it may offer the most significant advantages.

**Disclosure of Interest:**

None Declared

